# Effect of fruit intake on functional constipation: A systematic review and meta-analysis of randomized and crossover studies

**DOI:** 10.3389/fnut.2022.1018502

**Published:** 2022-10-06

**Authors:** Jinghong Huo, Lingyu Wu, Jinming Lv, Hongdou Cao, Qinghan Gao

**Affiliations:** ^1^School of Public Health and Management, Ningxia Medical University, Yinchuan, China; ^2^Key Laboratory of Environmental Factors and Chronic Disease Control, Ningxia Medical University, Yinchuan, China; ^3^Department of Neuroelectrophysiology, General Hospital of Ningxia Medical University, Yinchuan, China

**Keywords:** functional constipation, fruits, randomized and crossover studies, meta-analysis, gut microbiota

## Abstract

Functional constipation (FC) is commonly treated with fruits whose efficacy remains unclear. We conducted a meta-analysis of fruit intervention for FC and provided evidence-based recommendations. We searched seven databases from inception to July 2022. All randomized and crossover studies on the effectiveness of fruits on FC were included. We conducted sensitivity and subgroup analysis. A total of 11 studies were included in this review. Four trials showed that kiwifruits have significantly increased stool frequency (MD = 0.26, 95% CI (0.22, 0.30), *P* < 0.0001, I^2^ = 0%) than palm date or orange juice in the fixed-effect meta-analysis. Three high-quality studies suggested that kiwifruits have a better effect than ficus carica paste on the symptom of the FC assessed by the Bristol stool scale in the fixed-effect meta-analysis [MD = 0.39, 95% CI (0.11, 0.66), *P* < 0.05, *I*^2^ = 27%]. Besides, five trials showed that fruits can increase the amount of *Lactobacillus acidophilus* [MD = 0.82, 95% CI (0.25, 1.39), *P* < 0.05, *I*^2^ = 52%], analyzed with the random-effect model. Subgroup meta-analysis based on the types of fruits suggested that fruits including pome fruit, citrus fruit, and berries have increased the effect of *Bifidobacterium* t more than the stone fruits in the random effect meta-analysis [MD = 0.51, 95% CI (0.23, 0.79), *P* < 0.05, *I*^2^ = 84%]. Totally, fruit intake may have potential symptom alleviation on the FC as evidence shows that they can affect stool consistency, stool frequency, and gut microbiota. Further large-scale studies are needed to gain more confident conclusions concerning the association between fruit intake and FC in the future.

## Introduction

Constipation is a common functional bowel disorder, characterized by difficult, infrequent, or incomplete bowel movements (1). According to the Rome IV criteria, constipation is categorized into two subtypes: functional constipation (FC) and irritable bowel syndrome (IBS-C) (2). According to the Rome IV criteria in 2021, the global prevalence of FC was found to be 10.1% (3). In addition to its higher prevalence, chronic constipation comes with an economic burden for patients and health systems. Three million outpatient visits and 800,000 emergency room visits have been accounted for in the United States (4). The annual cost reached between $2,000 and $7,500 per patient in the United States in 2019 (5). Besides, the occurrence of constipation will also increase the poor quality of life, risk of colorectal cancer (6, 7), and higher rates of psychological distress (8). Therefore, it is necessary to emphasize the importance of successful prevention and management of constipation.

Until now, several common methods used to treat constipation have been applied in the clinic, including osmotic and stimulant laxatives, stool softeners, bulking agents, and pro-secretory agents. However, approximately half of the patients were dissatisfied with these treatment strategies due to the limited efficacy and side effects of drugs (9, 10). Dietary plays an important role in the treatment and management of constipation. The World Gastroenterology Association recommended increasing fiber intake either through dietary advice or supplementation (11). In the United Kingdom, professional guidelines in 2020 suggested that participants with constipation may consume fruits including prunes, cherries, and their fruit juices (12). Although epidemiological studies have also provided strong evidence that fruit could be beneficial in the FC, clinical trials showed inconsistent results. For example, a study with 1,088 participants including healthy and constipated patients suggested that some fruits, especially prunes, can soften the stool (13). But several trials in the clinic have found that some fruits, including ficus carica, palm date, and orange, did not affect the symptom alleviation of the FC, especially the stool consistency or frequency (14–16). In 2021, a recent systematic review of trials showed that various fruits, such as prunes, raisins, and apple fiber, could increase fecal weight. The present study suggests that apple, kiwifruit, fig paste, and orange may reduce gut transit time but prunes do not (17).

Therefore, this meta-analysis aims to evaluate the studies on the effect of fruits in patients with FC and to decide the fruit species that are most effective in treating participants with FC.

## Materials and methods

Our meta-analysis was conducted according to the Cochrane Handbook for Systematic Reviews of Interventions (18) and was performed based on the Preferred Reporting Items for Systematic Reviews and Meta-Analyses (PRISMA) statement (19). Two reviewers independently performed the literature search, study selection, data extraction, and quality assessment processes, such as the risk of bias and grading of evidence. Disagreements were resolved through discussion with the third author.

### Literature search

We aim to identify randomized and crossover studies through the following clinical research databases from their inception until July 7, 2022: PubMed, EMBASE, Web of Science, Chinese Biomedical Database (CBM), the Cochrane Library, the China National Knowledge Infrastructure (CNKI), and the China Science and Technology Journal Database. Combinations of keywords and medical subject headings (MeSH) terms as follows: “constipation,” “constipate,” “gut microbiome,” “gut transit,” “stool frequency,” “stool consistency,” “bowel movement,” “defaecation,” and “randomized controlled trials,” “crossover studies,” and “sorbitol,” “fruit,” “juice,” “fiber,” “polyphenol,” “extract,” “kiwi,” and “prune.” In addition, we manually searched the references of the original article and then reviewed relevant articles to find possible relevant studies.

### Selective criteria

Studies were eligible for inclusion if they met the following criteria: (1) The study was based on randomized controlled trials (RCTs) with a parallel or cross-over design in which fruit treatment was compared with placebo or no treatment; (2) The study population consisted of patients with functional constipation aged more than18 years; (3) The diagnosis of FC was clearly made by the use of internationally recognized criteria, such as Rome IV criteria; (4) The study used at least one of the following outcomes in clinical trials: stool consistency, stool frequency, Bristol stool score, *Lactobacillus acidophilus*, and *Bifidobacterium* spp. The following studies were excluded: (1) Case reports and reviews; (2) patients aged less than 18 years old; (3) patients with constipation who were induced by drugs or organic disease; (4) study protocols of ongoing trials without completed data.

### Study screening

**FIGURE 1:**
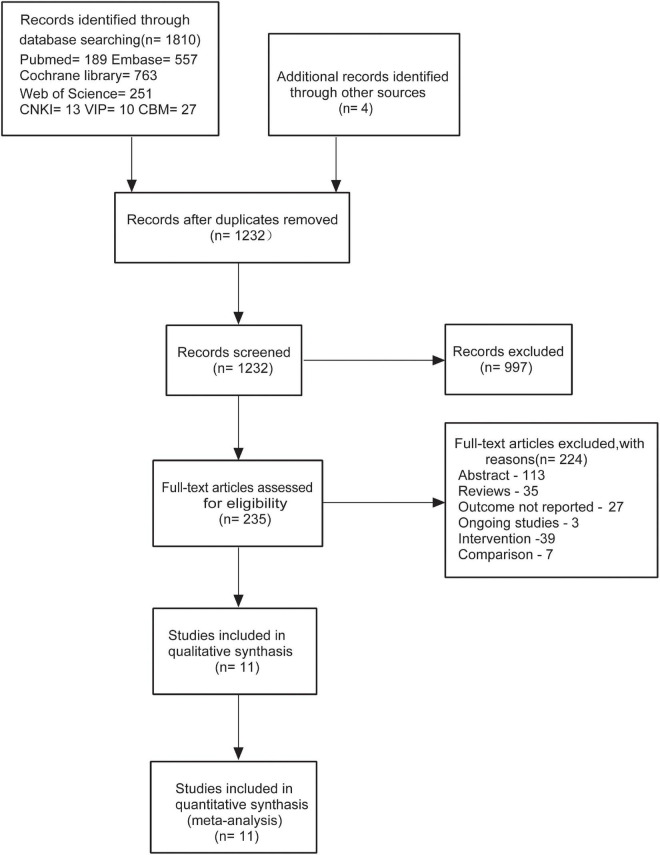
Flow diagram for the identification of relevant clinical trials examining the effect of fruits or fruit products on patients with functional constipation (FC).

Our meta-analysis was conducted independently by two reviewers. The screening was performed by three processes according to the inclusion and exclusion criteria. In the first stage, search results were downloaded from databases in EndNote and then duplicates were removed; and in the second stage, titles and abstracts of the articles were reviewed; in the last stage, the full text of studies where titles or abstracts that were insufficient to make decisions were obtained. The study screening diagram that suggested the detailed selection of studies is shown in Figure 1.

### Data extraction

Two reviewers independently extracted the data from the corresponding eligible studies, including the study design, first author’s name, year of publication, country of study, population (gender/age), duration of intervention, details of interventions (type, form, dosage), details of both the experimental treatment and the control and clinical outcomes (stool frequency, stool consistency, and gut microbiome) (Table 1).

**TABLE 1:** Summary of the human trials investigating the effect of fruits on functional constipation (FC).

Study	Country	Fruit product	Study design	Number	Study population	Daily dose	Comparator	Duration
Rush et al. (26)	New Zealand	Kiwifruit	Crossover RCT	48	Healthy adults	233 g	Placebo	3 weeks
Eid et al. (15)	UK	Palm date	Crossover RCT	22	Healthy adults	50 g	Maltodextrin	3 weeks
Shinohara et al. (25)	Japan	Apple	Crossover trials	8	Healthy adults	2 apples	Placebo	2 weeks
Chiu et al. (23)	China	Prune	RCT	60	Healthy adults	100 ml	Placebo drink	4 weeks
Lima et al. (16)	Brazil	Orange	Crossover trials	10	Healthy women	300 ml	Placebo	4 weeks
Mitsou et al. (24)	Greece	Banana	RCT	34	Healthy women	240 g	Placebo drink	8 weeks
Vendrame et al. (27)	Italy	Blueberry	Crossover RCT	20	Healthy male individual	250 ml	Placebo drink	6 weeks
Jamar et al. (28)	Brazil	Juçara	RCT	40	Individual with obesity	5 g	Maltodextrin	6 weeks
Baek et al. (14)	Korea	Ficus carica	RCT	80	Subject with FC	300 g	Placebo	8 weeks
Eady et al. (29)	New Zealand	Kiwifruits	Crossover RCT	32	Mildly constipated patients	3 kiwifruits	Metamucil	4 weeks
Wilkinson-Smith et al. (30)	UK	Kiwifruits	Crossover RCT	14	Healthy volunteers	300 g	Maltodextrin	3 days

### Risk of bias and grading of the evidence

We assessed the risk of bias in studies with the Cochrane Risk of bias tool for randomized trials version 2 (ROB2) (20). This tool suggests five detailed domains for the quality assessments of individual processes Five detailed domains that were assessed by two authors are as follows: (1) The randomization process; (2) the deviation from the intended intervention; (3) the missing results; (4) the measurement of the outcome; (5) the selection of the reported results. These domains were judged with high risks, some concerns, or low risk of bias judgments. In addition, for crossover trials, if the order of intervention was not randomized, the risk of bias in the randomization process was defined as high in the Cochrane tool. The bias due to carryover effects was evaluated by the process of a washout period or a follow-up non-interventional period (≥14 days) among the studies. Findings from these assessments have been summarized pictorially.

The Grading of Recommendations, Assessment, Development, and Evaluation (GRADE) tool was used to examine the quality and strength of the evidence (21). The evidence was graded as high, moderate, low, or very low quality. Although RCTs were graded as studies with high-quality evidence in any type of study, not all of the RCTs had higher quality due to various factors in the study design. It was, therefore, necessary to downgrade evidence with criteria and these included: study limitation (as assessed by the Cochrane ROB2), inconsistency (without unexplained heterogeneity between studies, *I*^2^ > 50% and *P* < 0.10), publication bias (significant evidence of small study effects), indirectness, and imprecision.

Two review authors evaluated the risk of bias with the Cochrane RoB2 and the outcome evidence with the GRADE tool independently, resolving any disagreements by a discussion with a third review author. We presented our assessment of the risk of bias and assessment of outcome evidence (Tables 2, 3; Supplementary Tables 1–5).

**TABLE 2:** Risk of bias assessment of randomized controlled trials of the effect of fruit intake on FC.

References	Random sequence generation	Blinding of participants and personnel	Incomplete outcome data	Measurement of outcome	Selective reporting
Chiu et al. (23)	Low	Some concerns	Low	Low	Low
Mitsou et al. (24)	Low	Some concerns	Low	Low	Low
Jamar et al. (28)	Low	Low	Low	Low	Low
Baek et al. (14)	Low	Low	Low	Low	Low

**TABLE 3:** Risk of bias assessment for crossover trials of the effect of fruits intake on FC.

References	Random sequence generation^a^	Blinding of participants and personnel	Incomplete outcome data	Measurement of outcome	Selective reporting	Carryover effects
Wilkinson-Smith et al. (30)	Low	Low	Low	Low	Low	Low
Vendrame et al. (27)	Low	Low	Low	Low	Low	Low
Eid et al. (15)	Low	Low	Low	Low	Low	Low
Eady et al. (29)	Low	Low	Low	Low	Low	Low
Lima et al. (16)	Low	Some concerns	Low	Low	Low	Low
Shinohara et al. (25)	Low	Some concerns	Low	Low	Low	Low

^a^For crossover studies, studies with “Some concerns” in the random sequence generation column were those that did not specify whether the order of treatments was randomized or not.

### Statistical analysis

Meta-analysis was conducted with Cochrane Collaboration’s Review Manager 5.3. The mean differences (MDs) and 95% confidence intervals (CIs) of the outcome data for all constipation symptoms were used for meta-analysis, provided that these symptoms were reported in at least three studies. The results of this meta-analysis were expressed as MDs and 95% CIs, which were calculated for continuous data. The χ^2^ ^2^ tested heterogeneity between studies and *I*^2^ suggested the degree of heterogeneity. The *I*^2^ value greater than 50% was considered as significant heterogeneity. If data were without significant heterogeneity, the fixed effects model was used for pooled analysis. If the data had significant heterogeneity, random effects model was used for pooled analysis. A *p* < 0.05 was considered statistically significant when we tested the pooled data.

When the *I*^2^ value was 50% or greater, possible reasons for heterogeneity were found according to the following methods: (1) Subgroup analysis was performed based on different outcomes, different types of intervention, and methodological quality; (2) A sensitivity analysis was conducted by repeating the analysis after sequential exclusion of one study at a time from the meta-analysis with more than 2 study comparisons to detect the stability of results. When the removal of a study changed the magnitude (by >10%), the significance, the direction of the association, or the evidence of heterogeneity, it was considered as having an influential effect.

Publication bias was evaluated by the funnel plot and Egger’s test. *P* < 0.05 was considered to be statistically significant. But we cannot explore sources of heterogeneity or publication bias because less than 10 study comparisons were included in each outcome analysis (22).

## Results

This meta-analysis includes a total number of 11 single RCTs with parallel or cross-over designs. According to the Cochrane RoB2, among the 11 studies, four studies (16, 23–25) in the current review has some overall concerns Of these, all studies have a bias due to deviations from intended interventions. Besides, the other seven studies (14, 15, 26–30) are assessed as having low risks. The assessments of risk of bias are reported in Supplementary Tables 2, 3. Besides, the GRADE system is used to rate the certainty of evidence according to its internationally recognized standard.

**FIGURE 2:**
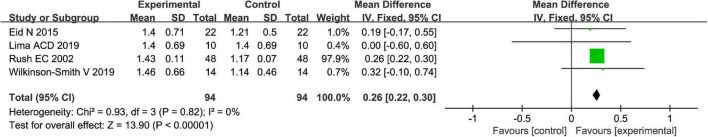
Effect of fruit intervention on stool frequency in the fixed-effect meta-analysis.

**FIGURE 3:**
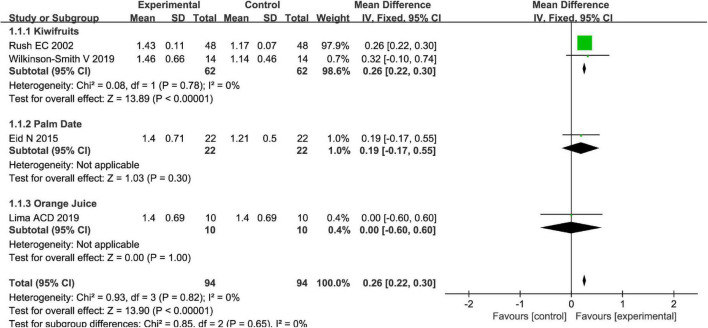
Intervention effect of different types of fruits vs. placebo for stool frequency on patients in subgroup meta-analysis.

Totally, fruits vs. placebo did significantly increase the stool frequency of patients with FC in the fixed-effect meta-analysis [MD = 0.26, 95% CI (0.22, 0.30), *P* < 0.00001, Figure 2]. There was no heterogeneity in this outcome of stool frequency (*I*^2^ = 0). Subgroup meta-analysis by the type of fruits suggested that kiwifruits have significantly increased stool frequency [MD = 0.26, 95% CI (0.22, 0.30), *P* < 0.0001, *I*^2^ = 0, Figure 3], while palm date or orange juice may not increase the stool frequency with the fixed-effect meta-analysis.

**FIGURE 4:**
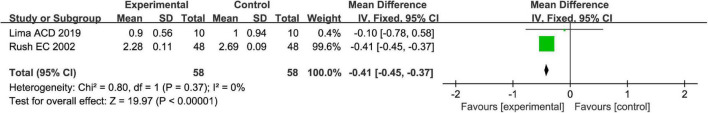
Effect of fruit intervention on stool consistency in the fixed-effect meta-analysis.

**FIGURE 5:**
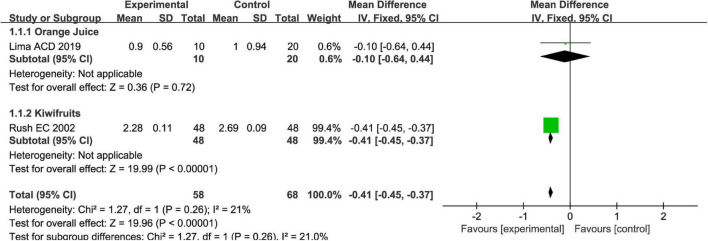
Intervention effect of different types of fruits vs. placebo for stool consistency on patients in subgroup meta-analysis.

Stool consistency is one of the main methods to measure symptoms of FC. Totally, fruits or fruit products vs. placebo have greater improvement in the stool consistency of patients with FC in the fixed-effect meta-analysis [MD = −0.41, 95% CI (−0.45, −0.37), *P* < 0.00001, Figure 4]. Kiwifruits have a greater symptom alleviation [MD = −0.41, 95% CI (−0.45, −0.37), *P* < 0.0001, Figure 5] than orange juice in the stool consistency of patients with FC by subgroup analysis.

**FIGURE 6:**
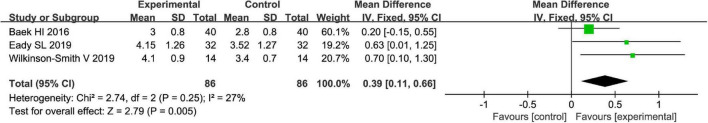
Effect of fruit intervention on the Bristol stool score in the fixed-effect meta-analysis.

**FIGURE 7:**
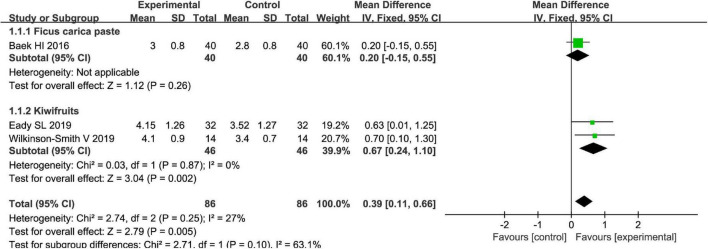
Intervention effect of different types of fruits vs. placebo for Bristol stool score on patients in subgroup meta-analysis.

The Bristol stool scale is used to assess the physical appearance and form of fecal samples. Totally, fruits were associated with beneficial effects on the physical appearance and form of fecal samples as evaluated by the Bristol stool scale [MD = 0.39, 95% CI (0.11, 0.66), *P* < 0.05, *I*^2^ = 27%, Figure 6]. However, a subgroup meta-analysis showed that kiwifruits have a better effect [MD = 0.67, 95% CI (0.24, 1.10), *P* < 0.05, Figure 7] than ficus carica paste on the symptom of the FC assessed by the Bristol stool scale in the fixed-effect meta-analysis.

**FIGURE 8:**
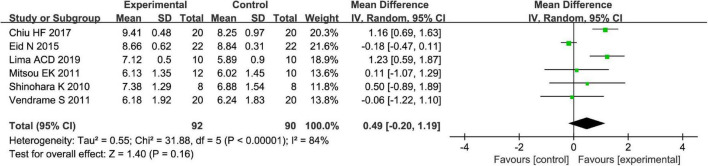
Intervention effect of different fruits vs. placebo for *Lactobacillus acidophilus* on patients.

**FIGURE 9:**
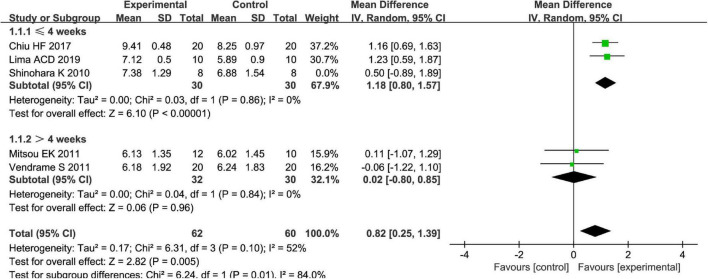
Intervention effect of different fruits vs. placebo for *L. acidophilus* on patients in subgroup meta-analysis by different intervention times.

Fruits vs. placebo have no effects on the *L. acidophilus* of patients with FC in the random-effect meta-analysis [MD = 0.49, 95% CI (−0.20, 1.19), *P* > 0.05, Figure 8]. There was high heterogeneity in *L. acidophilus* (*I*^2^ = 84%). A sensitivity meta-analysis by removing a trial suggested that heterogeneity has been decreased to 41% (Figure 9), and an analysis with a random-effect model showed that there are significant effects on *L. acidophilus* [MD = 0.81, 95% CI (0.31, 1.31), *P* < 0.05, Figure 9]. We also conducted a subgroup meta-analysis by intervention time on patients. Fruits affect the effect of L. *acidophilus* [MD = 1.14, 95% CI (0.77, 1.50), *P* < 0.05, Figure 9] when intervention time was ≤4 weeks. Analyzed with the subgroup meta-analysis, the heterogeneity among subgroups has been reduced to 0%. The difference between estimates of the effect of fruits on *L. acidophilus* in intervention time ≤4 weeks and intervention time >4 weeks was significant (χ^2^ = 5.81, *P* < 0.05 by a test of interaction; Figure 9).

**FIGURE 10:**
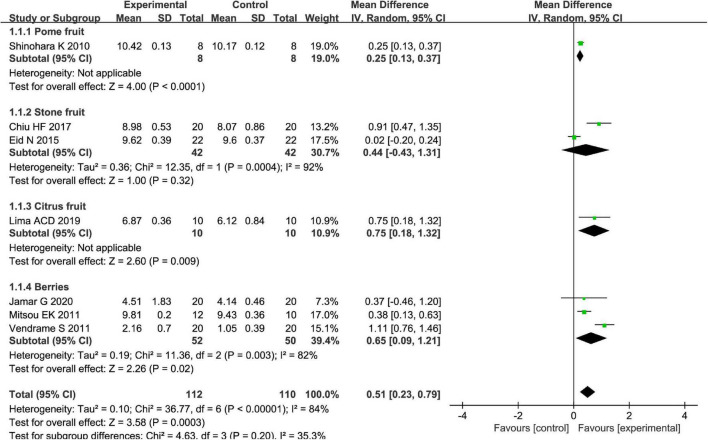
Intervention effect of different types of fruits vs. placebo for *Bifidobacterium* on patients in subgroup meta-analysis.

**FIGURE 11:**
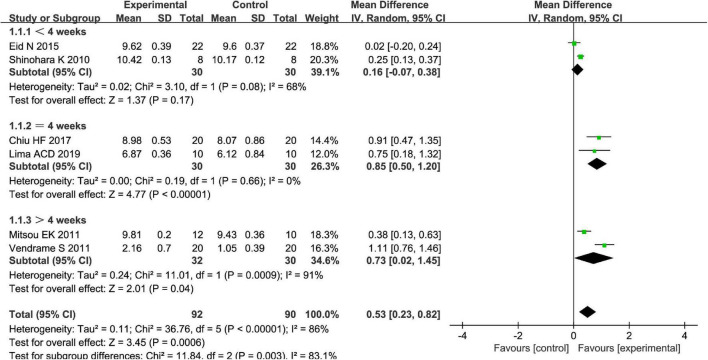
Intervention effect of the fruits vs. placebo for *Bifidobacterium* on patients in subgroup meta-analysis by types of intervention time.

Different types of fruits have various effects on the improvement of *Bifidobacterium* in patients. We performed a subgroup meta-analysis by the type of fruits for the *Bifidobacterium* and then suggested that fruits including pome fruits, citrus fruits, and berries have better effects on the *Bifidobacterium* than the stone fruits in the random effect meta-analysis [MD = 0.51, 95% CI (0.23, 0.79), *P* < 0.05, Figure 10]. Besides, we also conducted a subgroup meta-analysis by the intervention time where the effect of *Bifidobacterium* was increased both by ≥4 weeks and <4 weeks in the random effect meta-analysis [MD = 0.53, 95% CI (0.23, 0.82), *P* < 0.05, Figure 11].

## Discussion

The present meta-analysis showed that the consumption of fruits or fruit products was significantly associated with FC in the analysis of RCTs with parallel or cross-over design. The best of this study was the first to assess the different types of fruits in patients with FC in meta-analysis. These findings provided more support for the recommendations encouraging people to consume the most effective fruit to consume.

Fruit refers to the edible part of a plant that is, a mature ovary, consisting of seeds, covering, and any closely connected tissue. Fruit products are processed by fruits, such as frozen foods, canned food, juices, nectars, jams, and preserves. Our meta-analysis suggested that various fruits and fruit products have been shown to alter the microbiota and intestine motility in human studies, including kiwifruits (Bristol stool score, stool consistency, and bifidobacteria), blueberry (bifidobacteria), and orange (bifidobacteria). Kiwifruits are high in fiber and polyphenols and they contain vitamin C twice than orange. Pham et al. indicated that vitamin C can significantly increase microbial alpha diversity and fecal short-chain fatty acids, including butyrate and propionate, and the relative abundance of Collinsella (31). Kiwifruits have been studied for their effect on microbiota and intestine motility in *in vitro* experiments, animal studies, and human trials. Parkar et al. (32) suggested that kiwifruits produced high *Bifidobacterium* spp. compared to the control (water) in *in vitro* fermentation model (32). Various kiwifruits were investigated in animal trials, which shows that fruit components were able to increase the number of *Lactobacillus* spp. compared to the control (33, 34). In clinical trials on constipated adults, kiwifruits significantly increased bowel movement frequency in the constipated group but not in the healthy group (35, 36). Therefore, kiwifruits are more recommended to be consumed by patients with FC based on the current experiments, animal studies, and human trials.

Blueberries were popularized as a “super fruit” mainly due to abundant anthocyanin flavonoids (37). In an experiment with blueberries rich in anthocyanins on mice, gut microbiota, such as *Actinobacteria*, *Coriobacteriaceae*, and some members of *Bifidobacteriaceae*, were increased (38). A randomized crossover trial on adults suggested that the abundance of *Bifidobacterium* spp. and *L. acidophilus* increased compared to the baseline (27). To sum up, blueberry had positive effects on the microbiota composition, including *Bifidobacteria* and *L. acidophilus*. The content of fiber in oranges is higher than in other fruits, such as kiwifruits, apples, plums, and bananas. A randomized crossover trial involving adults has shown that the orange juice group had a higher abundance of the Porphyromonadaceae family and Parabacteroides genus, and the Odoribacteraceae family and Butyricimonas genus than placebo (39). However, we did not equate the effect of orange juice with orange due to lack of fiber in orange juice. So, we would like to suggest that fresh orange should be performed on the human clinical trials in the future.

Ideally, long-term randomized control trials would provide the strongest level of evidence for clinical guidelines. But these studies can be challenging to perform, especially for an intervention, such as fruits and fruit products. A randomized trial has suggested that kiwifruits may improve constipation symptoms in patients with constipation in 2021 (40), which is consistent with the results of our study that kiwifruits have significantly increased stool frequency [MD = 0.26, 95% CI (0.22, 0.30), *P* < 0.0001, *I*^2^ = 0]. Although our meta-analysis suggests that kiwifruit products have a potential symptom alleviation in constipation, the key is whether the kiwifruits planted in different countries have the same symptom-improving effect. We need to be cautious in deciding whether certain types of fruits are suitable or better than others for patients with constipation. A recent systematic review published in 2021 has suggested that there is some evidence for the effects of fruits on gut motility due to gut physiology and microbiota and are helpful in constipation symptom alleviation. However, it is hard to know the effects of fruits and the specific mechanisms behind their potential (17).

Several potential mechanisms have been suggested to explain the relationship between fruit consumption and chronic constipation. Increasing evidence from epidemiological studies in humans and experimental studies in animals showed that altered microbiota has been linked to constipation, and patients with constipation have unbalanced microbiota, such as *Bacteroidetes*, *Bifidobacteria*, and *Lactobacilli*, compared to patients without constipation (41–44). Therefore, lower beneficial microbiota is one of the major causes of constipation and regulating these microbiotas could be one of the major mechanisms.

Fruits are sources of sorbitol, polyphenols, and fiber (45), which are served as a core element of the “Five a Day” fruit recommendation by World Health Organization (WHO) (46). Sorbitol is a beneficial nutrient contained in fruits. Dietary sorbitol cannot be digested and absorbed and has the ability to hold water in its molecules (47, 48). Several studies have shown that sorbitol significantly increased fecal water or fecal weight and then eased constipation (49). It is well-known that polyphenols are inhibitors in fruits for the digestion of carbohydrates. Therefore, fruit intake containing polyphenols may increase undigested carbohydrates that are ready for fermentation by gut microbiota. In addition, the results found that 90–95% of ingested polyphenols reach the colon, which can affect gut microbiota composition and can be metabolized by gut microbiota (50). Some evidence showed that polyphenols have the ability to actively regulate the gut microbiota by increasing the bacteria, such as Bifidobacterium and Lactobacillus, that are helpful for gut health (51–53). While it has been emphasized that polyphenols would be beneficial in the improvement of inflammatory bowel disease as well IBS due to their anti-inflammatory ability (53), data without enough evidence have suggested a direct effect on constipation. Dietary fiber might also contribute to improvement in constipation by different potential mechanisms. Fiber, which is the sum of carbohydrates and it cannot be digested or absorbed in the small intestine, is characterized by polymers of three or more monomeric units (54). Non-fermentable fiber can enter the lower gut intact while viscous fibers have a potential water-binding ability, which can bulk stool significantly (55). Besides, gut microbiota abundance and fecal biomass can be increased by fermentable fiber intake, and the short-chain fatty acid production may also be increased (56).

We performed subgroup analysis to identify potential sources of heterogeneity. Within the subgroup analysis, we examined intervention time as a possible source of heterogeneity; this did show significant interaction between variables (χ^2^ = 11.84, *P* < 0.05 by the test of interaction, Figure 11). After analysis by the random-effect model, it is shown that an intervention time of fewer than 4 weeks may be better for Bifidobacterium of FC among four RCTs when the fruit intervention is compared with control in adults (16, 23, 24, 27).

Heterogeneity could also be explained by the differences between the studies of the method of dietary assessment. Vendrame et al. (27) collected data *via* self-completed food frequency questionnaires (27); five studies used 3-day dietary records (14, 15, 26, 28, 29) and three studies used medical questionnaires along with dietary habits (16, 23, 24), the remaining studies did not mention the dietary assessment (25, 30). Assessing true dietary intake is inherently difficult, and the use of food frequency questionnaires has been challenged (57, 58). They are likely to cause random or systematic errors. These measurements did not estimate the real connection between diet and diseases. So, we hope that there is a need to incorporate more biological markers of fruits, such as plasma vitamin C into nutritional assessment studies in future clinical trials. Besides, the polyphenols and polyphenol-rich whole foods may have a prebiotic function, with emphasis on the bifidogenic effect, leading to increased excretion of acetate Jamar et al. (28). Although acetate has the potential ability to be a microbiota metabolite, we would like to suggest that large-scale randomized control trials are needed to gain confident conclusions concerning the association between fruit intake and microbiota metabolites in future clinical research.

The current study has some strengths. We included higher-quality studies that have a low risk of bias and high validity for each study, and there are no significant baseline differences between the control and intervention groups. Besides, this is the first study to explore the relationship between fruits and constipation by meta-analysis.

Potential limitations should be considered. First, assessment of real dietary intake like food frequency questionnaires is inherently difficult, which cannot truly estimate the true interaction between fruit and constipation. It is very necessary to emphasize a call for standardization of nutritional epidemiology. We suggest using specific biomarkers of fruit to assess the dietary intake in future clinical trials. Besides, we had to admit that publication bias is a potential concern in the included studies because the statistical power may be limited since seven studies alone could not assess the publication bias for outcomes, such as *Bifidobacterium*.

## Conclusion

Our meta-analysis of randomized and crossover studies demonstrates that intake of fruits is linked to symptom alleviation of FC. Kiwifruits have significantly increased stool frequency than palm date or orange juice in the fixed-effect analysis. Pome fruit, citrus fruit, and berries have increased the *Bifidobacterium* than the stone fruits analyzed by the random-effect model. Prune and orange can increase the number of *L. s acidophilus* compared to the banana or blueberry analyzed with the random-effect model. Further, large-scale studies are needed to gain confident conclusions concerning the association between fruit intake and FC.

## Data availability statement

The original contributions presented in this study are included in the article/Supplementary material, further inquiries can be directed to the corresponding author.

## Author contributions

JH and LW: conceptualization and data curation. JL: formal analysis, investigation, and methodology. HC: supervision, validation, and visualization. QG: writing—original draft and writing—review and editing. All authors have read and agreed to the published version of the manuscript.

## Supplementary material

Supplementary Table 1 The effect of fruits on stool frequency of patients with FC.

Supplementary Table 2 The effect of fruits on the stool of consistency patients with FC.

Supplementary Table 3 The effect of fruits on Bristol stool score of patients with FC.

Supplementary Table 4 The effect of fruits for *Lactobacillus acidophilus* in patients with FC.

Supplementary Table 5 The effect of fruits for *Bifidobacterium* in patients with FC.
