# Supplementary figures and images for: Effect of fruit intake on functional constipation: A systematic review and meta-analysis of randomized and crossover studies
Source: Front Nutr. 2022 Oct 6;9:1018502. doi: 10.3389/fnut.2022.1018502 (PMC9583540; doi:10.3389/fnut.2022.1018502)

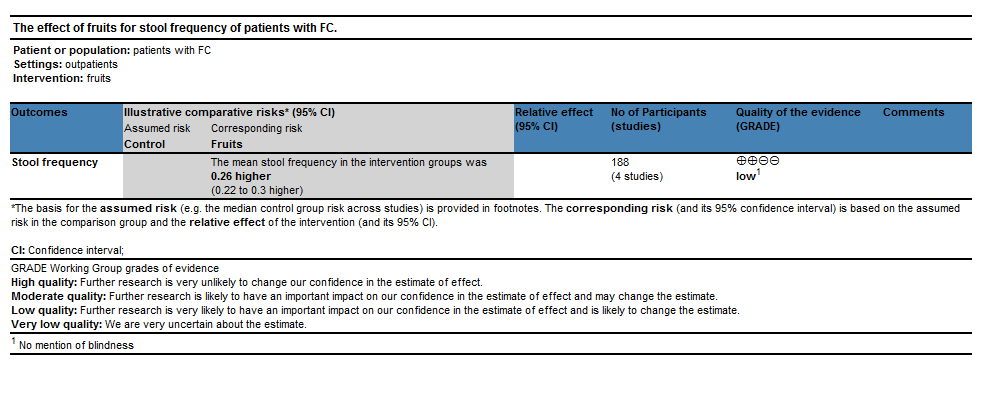

Supplement: Supplementary Table 1 — The effect of fruits on stool frequency of patients with FC. [file Data_Sheet_1.zip › Supplementary Table 1.PNG]

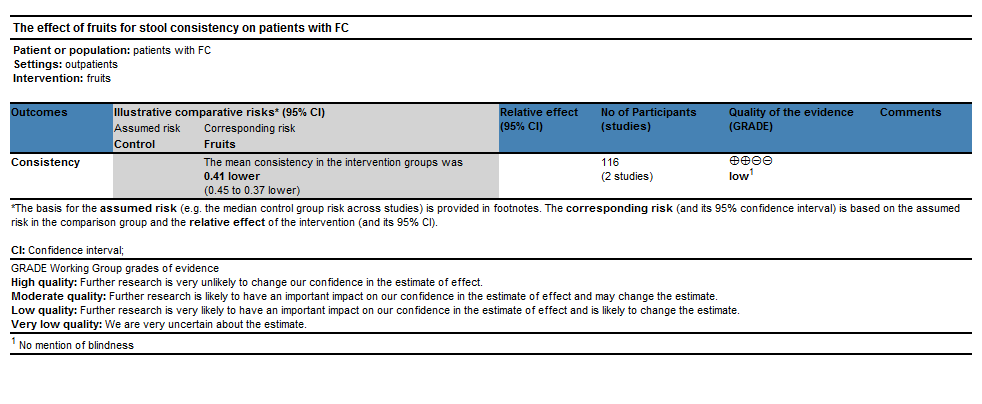

Supplement: Supplementary Table 1 — The effect of fruits on stool frequency of patients with FC. [file Data_Sheet_1.zip › Supplementary Table 2.PNG]

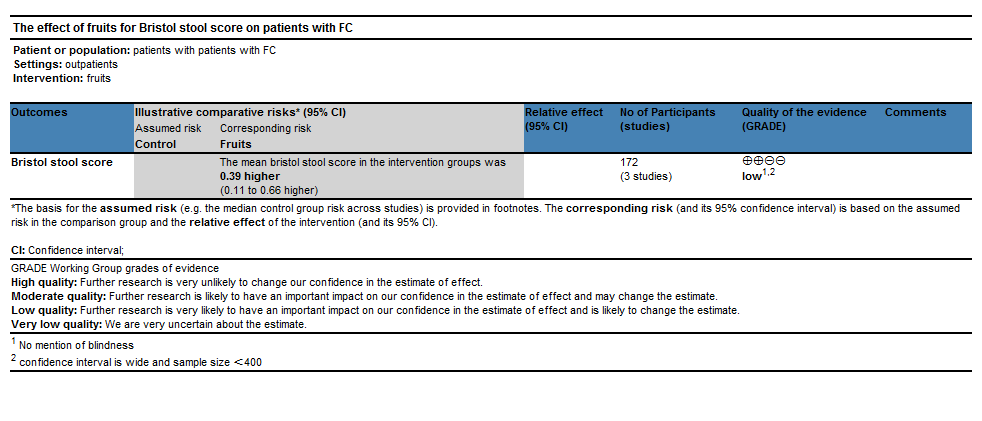

Supplement: Supplementary Table 1 — The effect of fruits on stool frequency of patients with FC. [file Data_Sheet_1.zip › Supplementary Table 3.PNG]

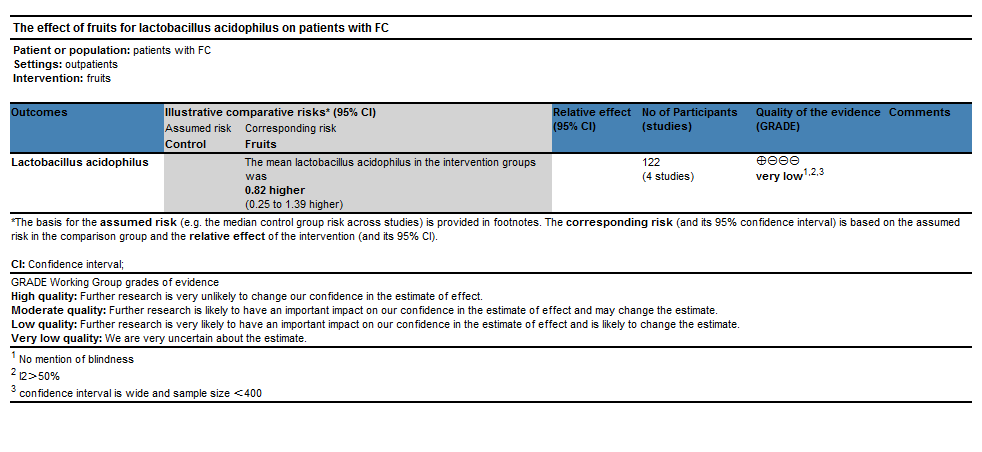

Supplement: Supplementary Table 1 — The effect of fruits on stool frequency of patients with FC. [file Data_Sheet_1.zip › Supplementary Table 4.PNG]

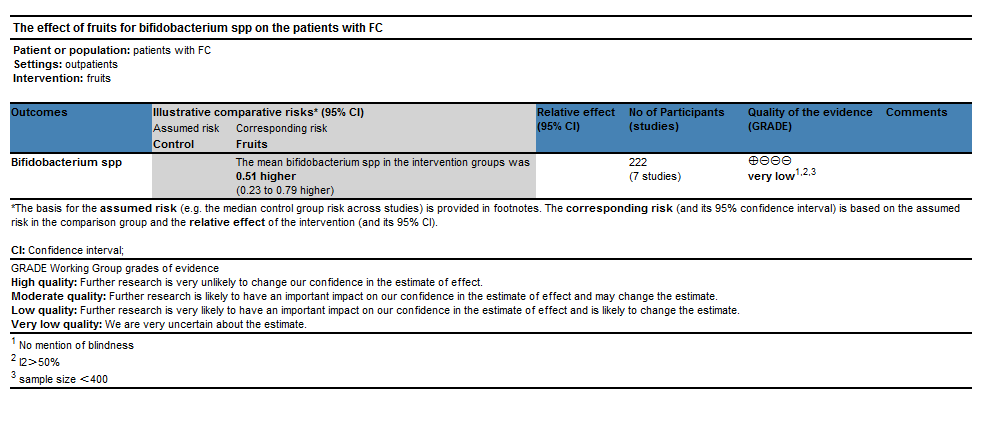

Supplement: Supplementary Table 1 — The effect of fruits on stool frequency of patients with FC. [file Data_Sheet_1.zip › Supplementary Table 5.PNG]
